# Sensitivity of primary fibroblasts in culture to atmospheric oxygen does not correlate with species lifespan

**DOI:** 10.18632/aging.100958

**Published:** 2016-05-07

**Authors:** Alison Patrick, Michael Seluanov, Chaewon Hwang, Jonathan Tam, Tanya Khan, Ari Morgenstern, Lauren Wiener, Juan M. Vazquez, Hiba Zafar, Robert Wen, Malika Muratkalyeva, Katherine Doerig, Maria Zagorulya, Lauren Cole, Sophia Catalano, Aliny AB Lobo Ladd, A. Augusto Coppi, Yüksel Coşkun, Xiao Tian, Julia Ablaeva, Eviatar Nevo, Vadim N. Gladyshev, Zhengdong D. Zhang, Jan Vijg, Andrei Seluanov, Vera Gorbunova

**Affiliations:** ^1^ Department of Biology, University of Rochester, Rochester, NY 14627, USA; ^2^ Laboratory of Stochastic Stereology and Chemical Anatomy (LSSCA), Department of Surgery, College of Veterinary Medicine and Animal Science, University of São Paulo (USP), São Paulo, Brazil; ^3^ School of Veterinary Medicine, Faculty of Health and Medical Sciences, University of Surrey, Guildford, Surrey, UK; ^4^ Science Faculty, Biology Department, Dicle University, 21280 Diyarbakır, Turkey; ^5^ Institute of Evolution, University of Haifa, Haifa 31905, Israel; ^6^ Division of Genetics, Department of Medicine, Brigham and Women's Hospital, Harvard Medical School, Boston, MA 02115, USA; ^7^ Department of Genetics, Albert Einstein College of Medicine, Bronx, NY 10461, USA

**Keywords:** fibroblasts, oxygen, senescence, human, rodents

## Abstract

Differences in the way human and mouse fibroblasts experience senescence in culture had long puzzled researchers. While senescence of human cells is mediated by telomere shortening, Parrinello et al. demonstrated that senescence of mouse cells is caused by extreme oxygen sensitivity. It was hypothesized that the striking difference in oxygen sensitivity between mouse and human cells explains their different rates of aging. To test if this hypothesis is broadly applicable, we cultured cells from 16 rodent species with diverse lifespans in 3% and 21% oxygen and compared their growth rates. Unexpectedly, fibroblasts derived from laboratory mouse strains were the only cells demonstrating extreme sensitivity to oxygen. Cells from hamster, muskrat, woodchuck, capybara, blind mole rat, paca, squirrel, beaver, naked mole rat and wild-caught mice were mildly sensitive to oxygen, while cells from rat, gerbil, deer mouse, chipmunk, guinea pig and chinchilla showed no difference in the growth rate between 3% and 21% oxygen. We conclude that, although the growth of primary fibroblasts is generally improved by maintaining cells in 3% oxygen, the extreme oxygen sensitivity is a peculiarity of laboratory mouse strains, possibly related to their very long telomeres, and fibroblast oxygen sensitivity does not directly correlate with species' lifespan.

## INTRODUCTION

Maximum lifespan varies greatly between mammalian species. Humans can live over 100 years, while mice, the most widely used research models, rarely live longer than 3 years, with most laboratory mice becoming ill by the age of two years. There are also multiple species of mammals with lifespans ranging from as short as 2 years in a shrew to 211 years in a bowhead whale [1]. Identifying physiological parameters that correlate with these drastic differences in lifespan can lead to insight into the molecular mechanisms of longevity.

Human and mouse primary fibroblasts are commonly used by biogerontologists to study cellular senescence. However, human and mouse cells behave very differently during routine culture [2]. Human fibroblasts undergo 50-70 population doublings and then enter an irreversible state of replicative senescence. Replicative senescence of human fibroblasts is triggered by telomere shortening, and these cells never spontaneously bypass senescence arrest. Mouse fibroblasts, in contrast, undergo only 10-15 population doublings and then enter a phase of slow growth, also referred to as senescence or crisis. However, after a few days, fast growing clones of mouse cells emerge and continue to proliferate. Mouse fibroblasts express telomerase and senescence in such cells is not related to telomere shortening. This difference between human and mouse cell behavior had caused a great deal of controversy in the field until Parrinello et al. [3] demonstrated that the slow proliferation of mouse fibroblast cultures results from a high sensitivity to atmospheric oxygen. Mouse cells grown in 3% oxygen proliferated faster and did not display the characteristic slow growth or senescence state as the same cells cultured in 21% oxygen. In addition, mouse cells in 21% oxygen accumulated more oxidative damage than human cells suggesting that the shorter-lived mice cannot efficiently counteract chronic oxidative stress.

Oxygen can be damaging to cells and tissues through the production of reactive oxygen species (ROS) during oxidative phosphorylation in the mitochondria. ROS react with and damage macromolecules such as enzymes, membrane lipids, and nucleic acids, thereby compromising the integrity of the cell. Early studies with cultured cells already noted that high oxygen levels slow down cell proliferation [4-6]. It was suggested that gradual accumulation of macromolecular damage due to ROS is the causal factor in aging [7, 8]. Studies of long-lived mutants with dampened insulin/IGF-1 signaling, such as C. elegans [9-12] and mice [13, 14], suggest that there is a correlation between resistance to oxidative stress and longevity. The finding that mouse cells are strikingly more sensitive to oxidative stress than human cells led to the hypothesis that the ability to prevent and/or repair oxidative damage contributes to the dramatic difference in the rate of aging between humans and mice [3].

To further investigate the possible correlation between oxygen sensitivity and aging, we compared the growth rate of primary fibroblasts under atmospheric (21%) and low oxygen (3%), conditions for 16 different rodent species whose maximum lifespans ranged from 4 to 32 years. In agreement with previous reports, fibroblasts derived from laboratory mice were highly sensitive to oxygen. However, fibroblasts from 9 other species of rodents and fibroblasts from wild-caught mice proliferated only slightly slower in atmospheric oxygen, while fibroblasts from 6 other species proliferated with the same rate in 3% and 21% oxygen. Hence, there was no correlation between species lifespan and oxygen sensitivity. These surprising results suggest that high oxygen sensitivity is a peculiarity of cells from laboratory mice, which possibly arose when wild mice were selectively bred to produce the common inbred laboratory strains.

## RESULTS AND DISCUSSION

To test whether sensitivity of fibroblasts to atmospheric oxygen correlates with lifespan, we cultured fibroblasts from 16 rodent species with lifespans ranging from 4 to 32 years in 3% and 21% oxygen. The list of species and their maximum lifespans are shown in Table 1. Primary fibroblasts were isolated from either skin or lung at 3% oxygen and frozen at PD<5. Only cells at low PD were used for the experiments to avoid selection of mutated clones. For each species, at least three cell lines, including both lung and skin fibroblasts, from three individual animals were examined. Lung and skin fibroblasts from the same species responded similarly to oxygen, therefore we pooled the results for lung and skin cells. Each cell line was taken out of liquid nitrogen storage, passaged once at 3% oxygen and then split into 3% and 21% oxygen conditions. Next, we compared the rate of growth under these conditions. We chose to compare the rate of growth rather than appearance of “senescence”, because the transient slowing of proliferation observed in mouse cells in 21% oxygen is highly variable among different cell lines.

**Table 1:** Maximum lifespan and body mass of the rodent species used in the study

Common name	Latin name	Maximum lifespan^1^, yr	Body mass^1^, g	Oxygen sensitivity^1^(3%O_2_ / 21%O_2_)
American beaver	*Castor canadensis*	24	20,250	1.30 ± 0.4
Blind mole rat	*Nannospalax ehrenbergi*	21	160	1.42 ± 0.05
Capybara	*Hydrochaeris hydrochaeris*	15	55,000	2.23 ± 0.46
Chinchilla	*Chinchilla lanigera*	17	640	0.81 ± 0.18
Deer mouse	*Peromyscus maniculatus*	8	20	0.98 ± 0.13
E. chipmunk	*Tamias striatus*	10	100	1.0 ± 0.09
E. grey squirrel	*Sciurus carolinensis*	24	530	2.23 ± 0.32
Golden hamster	*Mesocricetus auratus*	4	100	2.11 ± 0.31
Guinea pig	*Cavia porcellus*	12	730	1.15 ± 0.14
House mouse (laboratory)	*Mus musculus*	4	30	4.45 ± 0.52
House mouse (wild)	*Mus musculus*	4	30	1.63 ± 0.11
Mongolian gerbil	*Meriones unguiculatus*	6	50	1.05 ± 0.06
Muskrat	*Ondatra zibethicus*	10	1,360	1.42 ± 0.27
Naked mole rat	*Heterocephalus glaber*	32	35	2.02 ± 0.4
Norway rat (laboratory)	*Rattus norvegicus*	4	400	1.0 ± 0.08
Norway rat (wild)	*Rattus norvegicus*	4	400	1.02 ± 0.03
Paca	*Cuniculus paca*	16	9,000	1.52 ± 0.42
Woodchuck	*Marmota monax*	14	4,000	2.21 ± 0.32

1 The data was derived from AnAge database [28].

**Figure 1:**
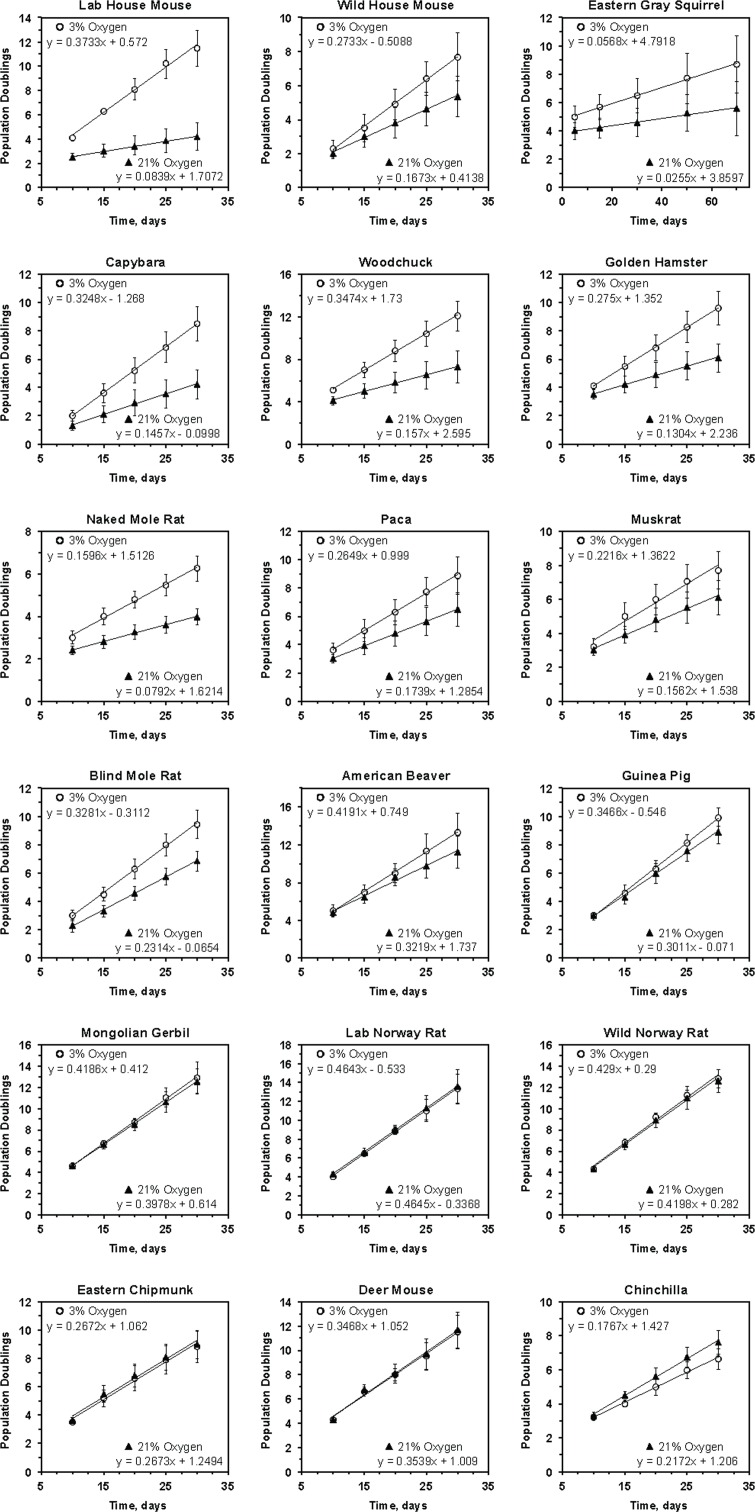
Growth rate of primary rodent fibroblasts in 3% and 21% oxygen The cells were cultured in either 3% or 21% oxygen atmosphere. The regression lines were fitted for the initial phase of linear growth, before the cultures became clonal. For each species the data is an average of at least 3 cultures, including both lung and skin fibroblasts, isolated from separate individuals, and error bars show s.d.

2 Oxygen sensitivity is calculated as the fibroblast growth rate at 3% O2 divided by the fibroblast growth rate at 21% O_2_, during the initial linear phase of growth ± STD (see Figure 1 for the growth curves).

In agreement with previous reports [3], we observed that cells derived from laboratory mice proliferated much slower (4.5-fold) in 21% oxygen than in 3% oxygen. Interestingly, cells isolated from wild-caught mice were markedly less sensitive to culture in atmospheric oxygen (Figure 1). Although the wild mouse cells proliferated slower in atmospheric oxygen the difference in growth rate was only 1.6-fold.

Cells from 9 species, including squirrel, capybara, woodchuck, hamster, naked mole rat, paca, muskrat, blind mole rat, and beaver, showed mild sensitivity to atmospheric oxygen, proliferating between 1.3- and 2-fold faster in 3% oxygen than in 21% oxygen. Cells from 6 species, including guinea pig, gerbil, rat, chipmunk, deer mouse, and chinchilla, showed no difference in proliferation rates between high and low oxygen conditions.

**Figure 2:**
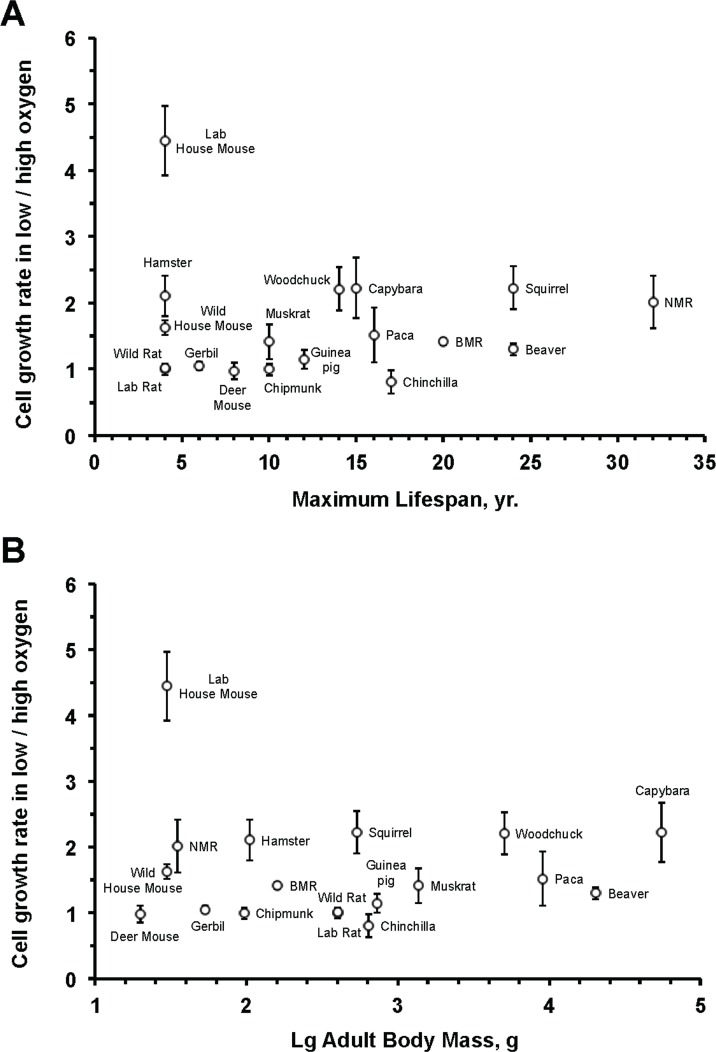
Fibroblast sensitivity to oxygen does not correlate with maximum lifespan (A) or body mass (B) The sensitivity to oxygen environment is reflected by the ratio between the growth rate at 3% and 21% oxygen. The rate of cell proliferation in 3% or 21% oxygen is the slope of the regression line shown in Figure 1. No significant correlation was observed between oxygen sensitivity and maximum lifespan (*r*^2^ = 0.0001; *P* = 0.97) or oxygen sensitivity and body mass (*r*^2^ = 0.006; *P* = 0.76). The correlation remained non‐significant after an outlier (laboratory mouse) was excluded: oxygen sensitivity and maximum lifespan (*r*^2^ = 0.135; *P* = 0.15); oxygen sensitivity and body mass (*r*^2^ = 0.075; *P* = 0.29).

To test whether oxygen sensitivity correlates with species lifespan, we calculated the slope of the initial logarithmic segment of the cell growth curve for each species under high and low oxygen conditions. The ratio between cell proliferation rates in low versus high oxygen showed no significant correlation to species maximum lifespan (*r*^2^ = 0.0001; *P* = 0.97) (Figure 2A) or body mass (*r*^2^ = 0.006; *P* = 0.76) (Figure 2B). The correlation remained non-significant after an outlier (laboratory mouse) was excluded: oxygen sensitivity and maximum lifespan (*r*^2^ = 0.135; *P* = 0.15); oxygen sensitivity and body mass (*r*^2^ = 0.075; *P* = 0.29) (Figure 2B).

With the exception of the house mouse, species in the oxygen-sensitive group tend to be long-lived. Thus, the slower growth in atmospheric oxygen for these species is unlikely to result from deficiency in their ability to repair oxidative damage, and may reflect more stringent cell cycle checkpoints. Human fibroblasts, similarly, show mild sensitivity to oxygen and tend to grow faster in 3% oxygen and can reach higher PDs prior to entering telomere-mediated senescence. These results suggest that resistance to oxidative stress is not necessarily associated with longevity, rather the cells from long-lived species may be more sensitive leading to more efficient elimination of damaged cells. Indeed higher resistance of human lung fibroblasts to oxygen has been associated with a disease condition [15].

Three of the species in the oxygen-sensitive group, capybara, paca and beaver, have a body mass greater than 8,000 g, their fibroblasts do not express telomerase activity and eventually enter telomere-mediated senescence [16, 17]. Two other species sensitive to oxygen, the naked mole rat and the blind mole rat, are very long-lived [18, 19], cancer-resistant [20-23], subterranean rodents. Considering their cancer resistance, these species are likely to have stringent cell cycle checkpoints. Furthermore, due to their sub-terranean lifestyle, these species are exposed to ambient oxygen concentrations lower than 21%, which may explain some of the sensitivity.

The species in the oxygen non-sensitive group, such as rat, gerbil, and deer mouse, tend to be shorter-lived. These animals may be better equipped than house mouse for counteracting oxidative damage and lack the stringent cell cycle checkpoints, resulting in identical growth rates in high and low oxygen.

Cells from wild-caught house mice were more resistant to 21% oxygen than cells from two laboratory mouse strains, C57BL/6 and 129/SvJ. This peculiar finding may be explained by the loss or silencing of certain antioxidant defense mechanisms in laboratory mice as a result of artificial selection and accumulation of deleterious alleles. An alternative explanation may be related to the telomere biology unique to laboratory mice. It has been shown [24] that laboratory mouse strains evolve much longer telomeres (∼100 kb), while the wild mice have more modest telomeres ranging between 10-30 kb in length. It is tempting to speculate that the very long telomeres of laboratory mice serve as a sink for oxidative damage during *in vitro* culture and result in the senescent phenotype displayed by mouse fibroblasts. Indeed, telomeric sequences are sensitive to oxidative stress [25] and damage to DNA in these sequences is repaired more slowly than the rest of the genome [26]. Another argument in support of this hypothesis is that laboratory rats do not exhibit longer telomeres than those of wild rats, and laboratory and wild rats do not differ in their sensitivity to oxygen (Figure 1). In conclusion, based on the analysis of multiple rodent species, the sensitivity of primary fibroblasts to culture in atmospheric oxygen does not significantly correlate with species lifespan.

## METHODS

### Animal samples

Capybaras were obtained from Bio Fau Assesoria e Comercio (São Paulo, Brazil). Pacas were from the animal facility at São Paulo State University. Outbred multicolored guinea pigs were purchased from Elmhill Labs. Chinchillas were purchased from Molton Chinchilla Ranch. Naked mole rats and blind mole rats were from the University of Rochester colonies. Two laboratory mice were C57BL/6, and one mouse was 129SvJ strain. Three mice (*Mus musculus*) were caught in a dormitory and cafeteria in New York state. Two rats were BN, two rats were F344, and three rats were wild caught in New York state. Outbred Mongolian gerbils Crl:Mon(Tum) and outbred hamsters Crl:LVG(Syr) were purchased from Charles River Laboratories. Beavers, deer mice, muskrats, chipmunks, woodchucks and squirrels were wild-trapped in New York state. All animals used in this study were young adults. Exact age was known for laboratory animals and was estimated for wild-caught animals from body measurements and fur color. Live animals were euthanized according to the University of Rochester Animal Care and Use Committee guidelines. Care was taken to minimize pain and discomfort to the animals.

### Tissue culture

Primary fibroblasts were isolated from lungs and under-arm skin as described [27]. The aliquots of cells were frozen in liquid nitrogen at PD<5. For the experiments, rodent fibroblasts were grown at 37°C and 3% CO_2_ in both high oxygen (21%) and low oxygen (3%) incubators on treated polystyrene culture dishes (Corning) using Eagle's Minimum Essential Medium (EMEM) supplemented with 15% FBS (Gibco) and 1% Penicillin-Streptomycin solution (Gibco). Cells were passaged regularly to avoid reaching confluency. During each passage, cells were counted using an automated cell counter and replated at 5×10^5^ cells/plate for all rodent cells, except naked mole rat which were plated at 2×10^5^ cells/plate.

### PD calculations

Using the cell count recorded at the time of passage, the number of times the plated cell population doubled (population doublings or PD) before being passaged was determined. This was calculated each time the cells were split using the following equation:

$${\text{PD}}_{\text{added}}=\log_{2}(\#\text{ cells counted}/\#\text{ cell plated})$$

In order to determine the number of times the original cell population (at the start of the experiment) had doubled at the time of each splitting, this PD was then added to the previous PD (PD_initial_) using the following equation:

$${\text{PD}}_{\text{final}}={\text{PD}}_{\text{initial}}+{\text{PD}}_{\text{added}}$$

The final PD (PD_final_) was then graphed against the number of days the cells had been growing in order to quantify cell growth rate.
